# Nationwide herd-level seroprevalence of *Neospora caninum* in dairy herds in Türkiye based on bulk tank milk ELISA and identification of associated risk factors

**DOI:** 10.3389/fvets.2026.1760253

**Published:** 2026-02-19

**Authors:** Mahmut Sinan Erez, Esma Kozan, Seydi Mehmet Arslan, Luis Pablo Hervé-Claude

**Affiliations:** 1 Department of Parasitology, Faculty of Veterinary Medicine, Afyon Kocatepe University, Afyonkarahisar, Türkiye; 2 Department of Clinical Sciences, Lewyt College of Veterinary Medicine, Long Island University - Post Campus, Brookville, NY, United States; 3 Department of Biomedical Sciences, Ross University School of Veterinary Medicine, Basseterre, Saint Kitts and Nevis

**Keywords:** abortion, bulk-tank milk ELISA, dairy cattle, herd-level prevalence, *Neospora caninum*, risk factors, vertical transmission

## Abstract

**Introduction:**

*Neospora caninum* is a major infectious cause of bovine abortion, leading to substantial reproductive and economic losses in dairy production systems worldwide. This study aimed to determine the nationwide herd-level prevalence of *N. caninum* in Türkiye and to identify farm-level risk factors associated with infection.

**Methods:**

Bulk-tank milk samples were collected from 466 dairy farms across 40 provinces representing seven geographical regions of Türkiye between June and November 2023. Antibodies against *N. caninum* were detected using a commercial competitive ELISA. Farm-level data on management practices, water sources, dog presence and feeding practices, pasture use, and abortion history were obtained through a structured questionnaire. Univariable and multivariable statistical analyses were performed to identify associations with herd-level seropositivity.

**Results:**

Overall herd-level seropositivity was 27.47% (128/466; 95% CI: 23.4–31.7%), with significant regional variation (*p* = 0.03394). The highest prevalence was observed in the Marmara (39.2%) and Southeastern Anatolia (37.5%) regions, while the lowest prevalence occurred in the Mediterranean region (16.7%). Herd seropositivity was strongly associated with reported abortion history, with seropositive herds having markedly higher odds of abortion (OR = 29.4; 95% CI: 16.5–52.9; *p* < 0.0001). Herds using groundwater had a significantly higher likelihood of seropositivity compared with those using municipal water (*p* = 0.002), an association that remained significant after multivariable adjustment. Although dogs were present on all farms, feeding practices varied regionally, with most farms providing leftover or raw animal-derived feed, while commercial dog feed use was largely restricted to western Türkiye.

**Discussion/conclusions:**

This study provides the first nationwide herd-level assessment of *N. caninum* exposure in Turkish dairy farms using bulk-tank milk ELISA and demonstrates widespread infection associated with key management and environmental risk factors. These findings support the implementation of targeted control strategies focusing on improved biosecurity, water hygiene, and routine herd-level monitoring to reduce vertical transmission and long-term infection pressure.

## 1 Introduction

Dairy cattle farming constitutes a major component of national milk production in Türkiye, where raw milk production increased by 4.7% in 2024 to 22,487,757 tons, of which 93.6% was derived from cows, placing Türkiye among the leading milk-producing countries in Europe and worldwide (1). Sustaining both the quantity and quality of milk production requires effective herd health management, as parasitic infections can reduce productivity, impair reproductive performance, and result in significant economic losses (39). *Neospora caninum*, an obligate intracellular protozoan parasite, is widely recognized as one of the leading infectious causes of bovine abortion worldwide, with substantial adverse effects on reproductive efficiency and herd profitability (2, 3). Current evidence indicates that, while human exposure has been reported, *N. caninum* is not regarded as a confirmed zoonotic pathogen.

Dogs and wild canids serve as the definitive hosts of *N. caninum*, while cattle and various other mammals act as intermediate hosts (2). Transmission occurs primarily through two routes. Horizontal transmission takes place when cattle ingest sporulated oocysts shed by infected canids, contaminating feed, water, or the environment. However, vertical (transplacental) transmission from dam to fetus is the dominant mechanism sustaining infection within herds and allows the parasite to persist across generations (4). Infected cows may abort, produce weak or stillborn calves, or give birth to clinically healthy but congenitally infected calves that remain infected for life and show an increased likelihood of abortion in subsequent pregnancies (5, 6).

Diagnosis in live animals relies primarily on serological detection of *N. caninum*-specific antibodies in serum or milk (7, 8). ELISA is widely used for herd-level surveillance because of its practicality and scalability. IgG1 antibodies are transferred into milk, and ELISA values obtained from milk and serum samples show strong linear correlation (9). Compared with individual serum sampling, bulk-tank milk ELISA enables efficient assessment of herd-level infection status using a single sample per farm, without animal handling, allowing faster and more cost-effective sample collection and feasibility at the national scale. Bulk-tank milk ELISA (BTM) therefore offers a minimally invasive and cost-effective tool for monitoring herd exposure, particularly suitable for large-scale epidemiological studies and routine herd-level screening (10–12).

Prevalence estimates for *N. caninum* vary widely between regions and production systems, influenced by environmental factors, herd management, and dog contact patterns (2, 3). In Europe, seroprevalence ranges from 16 to 76% in dairy herds and 41 to 61% in beef herds (13). In Türkiye, the mean animal level seroprevalence has been estimated at 14.7% based on individual cattle serum samples, similar to levels reported in several Asian and African countries (14). However, herd-level prevalence based on bulk-tank milk ELISA has not previously been reported in Türkiye.

Neosporosis also represents a substantial economic burden. Annual losses associated with *N. caninum* have been estimated at 546.3 million USD in the U. S. dairy industry and 9.7 million EUR in Swiss dairy herds (14, 15). For Türkiye, the economic impact has been estimated at approximately 710 USD per infected dairy cow, corresponding to national annual losses of 40.5 million USD (14). These estimates underscore the need for effective surveillance and management strategies.

Therefore, the objective of this study was to determine the nationwide herd level prevalence and spatial distribution of *N. caninum* in dairy cattle in Türkiye using bulk tank milk ELISA, and to identify management and environment related risk factors associated with infection. The findings aim to support the development of targeted control strategies to improve reproductive performance and reduce economic losses in the Turkish dairy sector.

## 2 Materials and methods

### 2.1 Study design

A cross-sectional study was conducted to determine the herd-level prevalence of *N. caninum* and to identify associated management- and environment related risk factors in dairy cattle across Türkiye. The study was carried out between June and November 2023 and covered all seven geographical regions of the country.

### 2.2 Sample size determination and farm selection

**Figure 1:**
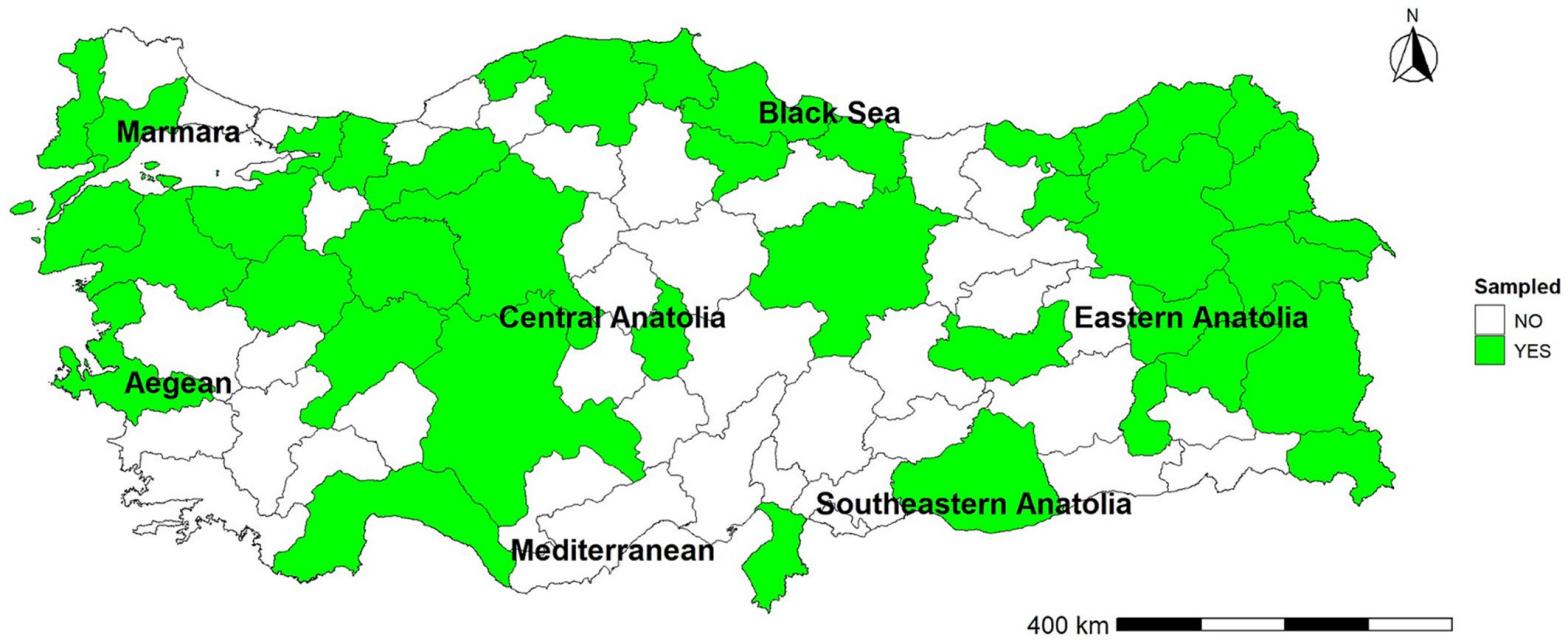
Provinces where milk samples were collected to determine *Neospora caninum* seroprevalence in bulk tank milk in 2023.

The target sample size was estimated for planning purposes using the standard formula for prevalence studies, assuming an expected herd-level prevalence of 20%, a 95% confidence level, and 5% absolute precision, which indicated a minimum of 246 dairy farms. To support regional coverage across Türkiye’s seven geographical regions and to allow stable region-level estimates, the target was conservatively increased to 466 farms to achieve proportional representation of major dairy-producing provinces. Dairy farms were enrolled using a non-randomized convenience sampling approach. Farmers were contacted through the extensive professional network of the main author, which primarily includes veterinarians from government and private sectors as well as practicing veterinarians, and additionally through a snowball sampling strategy (i.e., contacts of initially enrolled veteriarians or study participants). This approach enabled the inclusion of dairy farms from all seven geographical regions of Türkiye and across 40 provinces, achieving the calculated sample numbers and proportional regional representation. Although this sampling strategy has inherent limitations, the use of a non-randomized method was considered appropriate given the absence of a nationally available sampling frame for dairy farms (Figure 1). Inclusion criteria were: (i) being an active dairy enterprise, (ii) having at least 20 lactating cows, and (iii) being located within the predefined provinces selected to ensure regional representativeness.

### 2.3 Sample collection and questionnaire survey

From each farm, 50 mL of milk was collected directly from the bulk tank into sterile 50 mL Falcon tubes containing one tablet of sodium azide as a preservative. During sampling, standardized structured interviews were conducted with farm managers using a predefined questionnaire (Supplementary file S1). All interviews were administered by trained personnel to ensure consistency in data collection. Interviewers received standardized training through a dedicated in-person or online session during which the questionnaire was reviewed in detail and interview procedures were harmonized.

Information was collected on farm demographics and known risk factors for neosporosis, including water source, pasture use, presence and feeding practices of on farm and stray dogs, access of dogs to feed storage or grazing areas, and reported abortion history within the herd.

### 2.4 Processing and storage of bulk tank milk samples

All milk samples were transported to the laboratory under cold-chain conditions and processed immediately. Samples were centrifuged at 1,200 × g for 15 min in a temperature-controlled centrifuge set at +4 °C. The fat layer was removed using a sterile spatula, and the skimmed milk fraction was collected with a Pasteur pipette, aliquoted into 2 mL microcentrifuge tubes, and stored at −20 °C until serological analysis.

### 2.5 Serological analysis

Detection of *N. caninum*-specific antibodies in milk was performed using a commercial competitive ELISA kit (*Neospora caninum* Milk Competitive ELISA, IDVet, Grabels, France), according to the manufacturer’s instructions. Milk samples were thawed at room temperature prior to analysis. For each assay, 100 μL of milk sample, positive control, and negative control were added to the wells of the test plate and incubated. Plates were washed three times using the kit wash buffer, followed by the addition of 100 μL conjugate and incubation for 30 min at room temperature. After a further three washes, 100 μL substrate solution was added and incubated for 15 min in the dark. The reaction was stopped with 100 μL stop solution, and optical density (OD) was measured at 450 nm.

Results were expressed as the sample-to-negative control ratio (S/N%) calculated as:


$$S/N(\%)=(\frac{{\mathrm{OD}}_{\text{sample}}}{{\mathrm{OD}}_{\text{negative control}}})\times 100$$

Samples with S/N > 20% were considered positive, and those with S/N ≤ 20% were classified as negative, according to the manufacturer’s interpretation criteria.

### 2.6 Statistical analysis and model construction

Associations between *N. caninum* herd-level seropositivity and geographical region were evaluated using the Chi-square test. Associations with reported abortion history were assessed using contingency table analyses, with Fisher’s exact test applied when expected cell counts were <5. Odds ratios (OR) with 95% confidence intervals (CI) were calculated. When overall regional differences were statistically significant, *post hoc* pairwise comparisons between regions were conducted using Chi-square tests with *p*-values adjusted for multiple testing using the Bonferroni correction to control the family-wise error rate.

Multivariable risk factor analysis was performed using logistic regression, with herd-level *Neospora* serostatus as the binary outcome. Explanatory variables considered included water supply type (municipal, groundwater, spring), pasture proportion (<25%, 25–50, >50%), herd composition (number of cows and young stock), and dog feeding practices (feeding of raw or leftover animal-derived materials and scavenging versus supplementation with commercial dog feed). Due to high collinearity between the number of cows and young stock (Pearson *r* = 0.95), a combined herd size variable was used. Model selection was guided by backward elimination and biological plausibility.

The final model retained water supply, pasture proportion, and herd size as predictors. Adjusted ORs with 95% CIs were estimated, and statistical significance was assessed using Wald tests. Model fit was evaluated using Akaike Information Criterion (AIC), pseudo-R^2^, and likelihood ratio tests. Potential interactions between predictors were examined but did not improve model fit and were therefore excluded.

Multivariable modeling, data preprocessing, and diagnostic checks were performed using Python 3.12 (pandas and statsmodels), with figures generated using matplotlib and seaborn. Descriptive and univariable analyses were conducted in R (RStudio version 2023.06.0).

## 3 Results

### 3.1 *Neospora caninum* herd-level prevalence across regions

**Figure 2:**
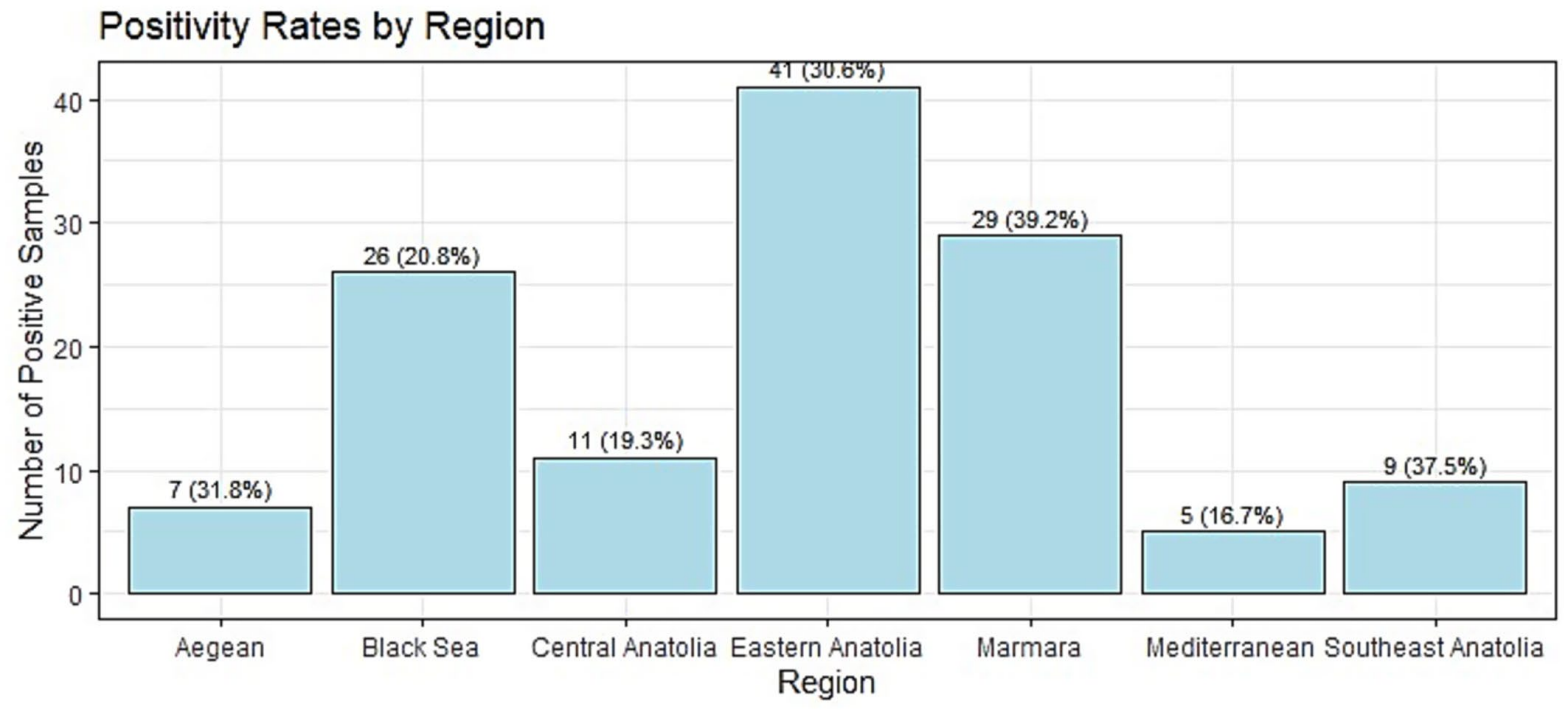
Percentage distribution of *Neospora caninum*-positive milk samples by region in a cross sectional study on bulk tank milk in 2023.

Bulk tank milk samples collected from 466 dairy farms across seven regions of Türkiye revealed an overall herd-level seroprevalence of 27.47% (128/466). Regional prevalence ranged from 16.7% in the Mediterranean region (5/30; 95% CI: 7.3–33.6%) to 39.2% in the Marmara region (29/74; 95% CI: 28.9–50.6%). The Aegean (31.8%; 7/22; 95% CI: 16.4–52.7%), Eastern Anatolia (30.6%; 41/134; 95% CI: 23.4–38.8%), and Southeastern Anatolia (37.5%; 9/24; 95% CI: 21.2–57.3%) regions also showed relatively high seropositivity, whereas lower rates were found in the Black Sea (20.8%; 26/125; 95% CI: 14.6–28.7%) and Central Anatolia (19.3%; 11/57; 95% CI: 11.1–31.3%) regions (Figure 2). Regional differences were significant (Chi-square: χ^2^ = 13.639, df = 6, *p* = 0.034). *Post hoc* pairwise comparisons between regions were not significant after adjustment for multiple testing (all adjusted *p*-values ≥ 0.17).

### 3.2 Association between *Neospora caninum* seropositivity and abortion history

The relationship between herd seropositivity and reported abortion history was evaluated using a contingency analysis based on ELISA S/N status (≥20% = positive). A significant association was identified (χ^2^ = 198.64, df = 1, *p* < 0.0001) and was also supported by Fisher’s exact test (*p* < 0.0001). The odds of reported abortion were higher in *N. caninum* positive herds than in seronegative herds (OR = 29.4; 95% CI: 16.5–52.9).

### 3.3 Water sources used in dairy production

Three primary water sources were reported: spring water (53.9%), groundwater (37.4%), and municipal (public) supply (6.1%) (Table 1). Spring water use was highest in the Black Sea (87.2%) and Eastern Anatolia (85.1%) regions, whereas groundwater was most frequent in Central Anatolia (84.2%) and the Aegean region (77.3%). Municipal water use was <27% in all regions and was highest in the Mediterranean region (26.7%). Water source was associated with *N. caninum* serostatus (χ^2^ = 12.331, df = 2, *p* = 0.002).

**Table 1:** Water sources used in dairy cattle farms across regions in Türkiye participating in a cross sectional study on bulk tank milk *Neospora caninum* prevalence in 2023.

Region	Groundwater (*n*, %)	Public supply (*n*, %)	Spring water (*n*, %)
Aegean	17 (77.3%)	3 (13.6%)	2 (9.1%)
Black Sea	14 (11.2%)	2 (1.6%)	109 (87.2%)
Central Anatolia	48 (84.2%)	9 (15.8%)	–
Eastern Anatolia	20 (14.9%)	–	114 (85.1%)
Marmara	48 (64.9%)	7 (9.5%)	19 (25.7%)
Mediterranean	18 (60.0%)	8 (26.7%)	4 (13.3%)
Southeast Anatolia	14 (58.3%)	–	10 (41.7%)
Total	179 (37.4%)	29 (6.1%)	258 (53.9%)

### 3.4 Dog presence and feeding practices

All surveyed farms (100%) reported the presence of dogs and confirmed that dogs had access to feed storage areas and that stray dogs were present in grazing pastures. Most farms (95.9%, *n* = 447) reported feeding dogs leftover food, raw meat, or allowing scavenging/hunting, while 4.1% (*n* = 19) reported supplementation with commercial canned or dry feed (in addition to the above-listed feed sources). Farms using commercial dog feed were located in western Türkiye.

### 3.5 Regional variation in pasture use

**Figure 3:**
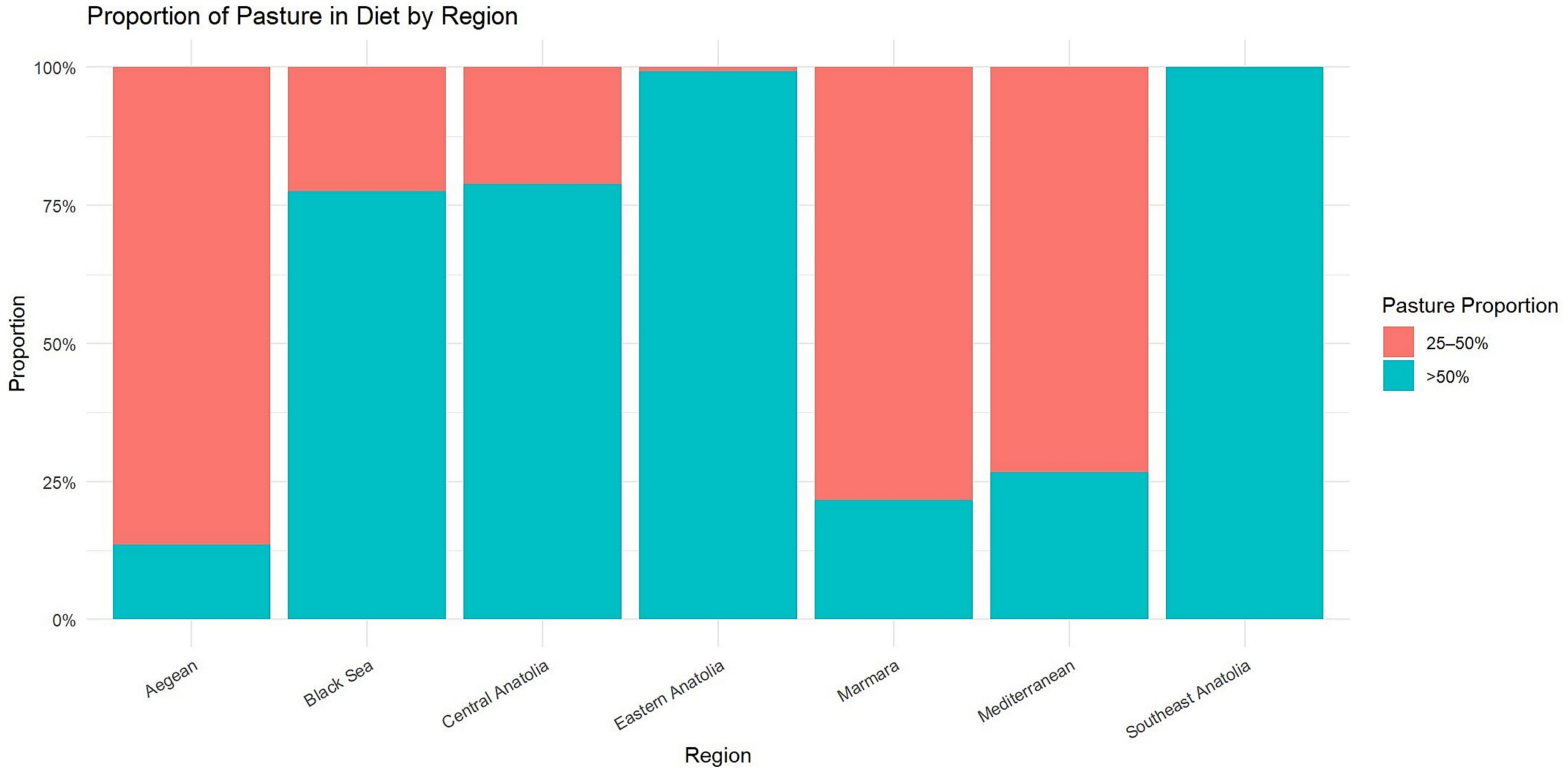
Regional variation in the contribution of pasture to the total diet of dairy cattle in farms across regions in Türkiye participating in a cross sectional study on bulk tank milk *Neospora caninum* prevalence in 2023.

The proportion of pasture in the total diet of dairy cattle varied significantly between regions (χ^2^ = 212.91, df = 6, *p* < 0.001). Pasture-based feeding (>50% of diet) predominated in Eastern Anatolia (99.3%), Central Anatolia (78.9%), and the Black Sea region (77.6%). In contrast, farms in the Aegean (86.4%), Marmara (78.4%), and Mediterranean (73.3%) regions relied more heavily on concentrate-based feeding (pasture constituting only 25–50% of the diet). All farms sampled in Southeastern Anatolia reported pasture use greater than 50% (Figure 3). In univariable testing, pasture proportion was not associated with *N. caninum* serostatus (Fisher’s exact *p* = 0.497).

### 3.6 Model results

Different models were explored considering all relevant variables in the study. Variables with no variability were removed. Variables with biological plausibility were retained regardless (pasture use, water sources, etc.). In the final multivariable logistic regression model, water supply and pasture exposure were retained as key predictors, along with herd size as a proxy for farm scale. The model demonstrated a pseudo-R^2^ of 0.029 and an AIC of 540.99, indicating moderate explanatory power while maintaining parsimony. Farms using groundwater had significantly higher odds of *N. caninum* positivity (OR = 4.77, 95% CI: 1.38–16.41, *p* = 0.013) compared to those using municipal water. Spring water showed a non-significant trend (OR = 2.33, 95% CI: 0.66–8.13, *p* = 0.187). Pasture exposure >50% was associated with increased odds (OR = 1.45, 95% CI: 0.87–2.42, *p* = 0.149), though not statistically significant. Herd size did not demonstrate a meaningful effect (OR = 0.99, 95% CI: 0.98–1.02, *p* = 0.873) (Table 2).

**Table 2:** Logistic regression findings when exploring risk factors for *Neospora caninum* seropositivity in bulk tank milk in Turkish farms in 2023.

Predictor	Category	OR	95% CI	*p*-value
Water supply	Municipal	REF		
	Groundwater	4.77	1.38–16.41	0.013*
	Spring water	2.33	0.66–8.13	0.187
Pasture %	25–50% pasture	REF		
	>50%	1.45	0.87–2.42	0.149
Herd size (per animal)		0.99	0.98–1.02	0.873

^*^Statistically significant result at *p* < 0.05.

## 4 Discussion

*Neospora caninum* is widely recognized as a major infectious cause of reproductive loss in dairy cattle worldwide, with substantial economic consequences for the cattle industry (6). In the present study, a herd-level seroprevalence of 27.47% was detected across 466 dairy farms in Türkiye, demonstrating that exposure to *N. caninum* is widespread at the national level. Previous serological studies conducted in Türkiye, primarily based on individual serum samples, have reported highly variable prevalence estimates across regions (16–19). Such variability may partly reflect Türkiye’s pronounced climatic heterogeneity, ranging from humid and temperate coastal areas to continental and semi-arid inland regions, which can influence oocyst survival, environmental contamination, and exposure risk. In this context, bulk-tank milk (BTM) ELISA represents a practical and scalable herd-level surveillance tool, enabling assessment of infection status with reduced sampling effort and cost and allowing repeated monitoring across large production systems (10, 20). Importantly, to date, no nationwide herd-level evaluation of *N. caninum* exposure based on BTM ELISA had been conducted in Türkiye. The prevalence observed in this study aligns with global estimates, being comparable to reports from Italy (30.7%) (21), higher than those from Sweden (8.3%) (22) and Alberta, Canada (7.4–18.2%) (23), and lower than values reported from Iran (55%) (24), situating Türkiye within the broader international epidemiological context.

Marked regional variation in herd-level seropositivity was observed, with the highest prevalence recorded in the Marmara Region (39.2%), followed by Southeastern Anatolia (37.5%) and the Aegean Region (31.8%), while the lowest prevalence was observed in the Mediterranean Region (16.7%). These regional differences likely reflect variation in herd management practices, the density and behavior of domestic and stray dogs, grazing intensity, and local environmental conditions that influence oocyst persistence and transmission. Comparable geographic patterns have been documented in European and Asian production systems, where dog contact and grazing management are recognized as key determinants of exposure risk (25–27). Although overall regional heterogeneity was statistically significant, the absence of significant pairwise contrasts after adjustment suggests that these differences reflect broad regional trends rather than discrete, isolated hotspots of infection.

A strong association was identified between herd seropositivity and abortion history. *N. caninum* positive herds had 29.4 times higher odds of reporting abortion than seronegative herds, reinforcing the well-established role of *N. caninum* as a leading cause of infectious abortion in dairy cattle (28–30). Similarly, a large-scale study involving over 3,200 herds in Rhineland-Palatinate, Germany, reported that bulk tank milk–positive herds experienced an annual abortion rate approximately 3% higher than negative herds, further confirming the herd-level reproductive impact of *N*. *caninum* (10). Previous research has also demonstrated that herds with high BTM antibody levels may incur productivity losses of approximately 1.6 kg of milk per cow per day (9). Collectively, these findings emphasize that *N. caninum* infection contributes not only to reproductive loss but also to reduced herd-level performance and economic efficiency.

Risk factor analysis further underscored the importance of environmental and management-related transmission pathways. In particular, the use of groundwater was associated with a significantly higher likelihood of herd seropositivity compared with farms relying on municipal water supplies. This association supports the hypothesis that untreated or naturally sourced water may serve as an important environmental transmission route, potentially through contamination with oocysts shed in dog feces (31, 32). Although dogs were present on all surveyed farms, notable regional differences were observed in dog feeding practices. The majority of farms (95.9%) reported feeding dogs leftover household food, raw meat, or allowing scavenging and hunting behavior, whereas supplementation with commercial dog feed was confined to a limited number of farms in western Türkiye. Feeding practices involving raw or scavenged animal tissues increase the likelihood of exposure to infective tissue cysts and may therefore contribute to sustained parasite circulation on farms (33). Comparable associations between *N. caninum* seropositivity and environmental or management-related factors, including water source and production system, have also been reported in recent studies of small ruminants in Mexico and southeastern Türkiye, underscoring the broader relevance of these transmission pathways across livestock systems (34, 35).

In addition to horizontal environmental exposure, vertical (transplacental) transmission represents the dominant mechanism sustaining *N. caninum* infection within cattle populations (2). Through this route, infection can persist across multiple generations even when dog-mediated environmental contamination is reduced. In the absence of effective treatment options to prevent abortion or congenital infection, control strategies must therefore prioritize interruption of vertical transmission, particularly through selective breeding practices such as avoiding the use of seropositive cows as replacement breeders. Nevertheless, horizontal transmission remains epidemiologically relevant. Dog presence and local dog density have been identified as important drivers of elevated BTM seroprevalence (12), and although the present study did not capture variability in dog presence, it confirmed a consistent presence of dogs on dairy farms, often with unrestricted access to pastures and feed storage areas. Accordingly, complementary control measures should include restricting canine access to placental tissues and feed materials and ensuring appropriate disposal of aborted tissues (36). Over time, the combined implementation of these measures may contribute to a gradual reduction in herd antibody levels and overall infection pressure (37).

The relevance of dog-mediated transmission is further amplified in the Turkish context by the widespread presence and high density of stray dogs across both rural and peri-urban landscapes. Recent ecological evidence from Türkiye has demonstrated extensive spatial and temporal overlap between stray dogs and native wildlife species, highlighting the capacity of free-ranging dogs to move across fragmented ecosystems and interact with multiple hosts (38). Such unrestricted movement increases the likelihood of environmental contamination with *N. caninum* oocysts, particularly in shared grazing areas, water catchments, and feed-access zones used by livestock. Given the scale and persistence of stray dog populations in Türkiye, their role in sustaining environmental transmission cycles should be considered a critical component of neosporosis epidemiology and control strategies at the national level.

Pasture use varied significantly between regions; however, the proportion of pasture in the diet did not show a statistically significant association with seropositivity after multivariable evaluation. This may partly reflect differences in seasonal grazing practices, as pasture access in many regions is limited to specific periods of the year, potentially reducing sustained exposure to contaminated environments. In contrast, drinking water sources are used continuously throughout the year and therefore appear to represent a more consistent risk factor for *N. caninum* exposure.

The findings of this study align with national economic estimates indicating that neosporosis represents a substantial financial burden in Türkiye, with an estimated cost of approximately 710 USD per infected dairy cow and total annual industry losses of 40.5 million USD (14). Identifying region-specific risk profiles and modifiable management practices is therefore essential for designing cost-effective control strategies.

The present findings indicate that *N. caninum* exposure is widespread among dairy herds in Türkiye and that herd-level seropositivity is associated with specific management- and environment-related factors.

### 4.1 Study limitations

The use of non-randomized samples in large veterinary farm based studies is commonplace in resource scarce settings, resulting in the lack of a farm sampling frame. This type of sampling has the potential of introducing bias, as there is no reliable way to identify if farms were selected by an underlying charcateristic that might impact the probability of being positive (or negative) to *N. caninum*. Future regional studies, in a reduced geographical setting might be constructed with a random design to control for some of the bias that we might have encountered in this study.

Furthermore, the cross-sectional study design limits causal inference (e.g., abortions and *N. caninum* seropositivity), and bulk-tank milk ELISA does not allow differentiation between chronic, recrudescent, or recent infections. In addition, information on abortion history and management practices was obtained through farmer questionnaires and may therefore be subject to recall bias and potential misclassification. Some variables showed limited or no variation across farms, reducing their informativeness for inclusion as risk factors in the multivariable model. Nevertheless, the large sample size and broad geographical coverage strengthen the generalizability of the findings. Future studies incorporating longitudinal designs and refined questionnaire approaches may help to further elucidate the role of farm and stray dogs in *N. caninum* transmission in Turkish dairy systems.

## 5 Conclusion

Based on the risk factors identified, control strategies should prioritize preventing dog access to feed storage and calving areas, improving disposal of placental tissues and aborted materials, minimizing the use of untreated water sources where feasible, and implementing routine bulk-tank milk monitoring to guide herd-level intervention. Importantly, because vertical transmission is the primary mechanism maintaining infection within herds, management programs should also consider avoiding the selection of seropositive cows as replacement breeders to prevent perpetuation of infection across generations. Future work should include longitudinal follow-up to clarify transmission dynamics and integrate molecular genotyping to support the development of targeted and regionally adapted control strategies.

## Data Availability

The original contributions presented in the study are included in the article/Supplementary material, further inquiries can be directed to the corresponding author.
