# Supplementary material for: Nationwide herd-level seroprevalence of Neospora caninum in dairy herds in Türkiye based on bulk tank milk ELISA and identification of associated risk factors
Source: Front Vet Sci. 2026 Feb 19;13:1760253. doi: 10.3389/fvets.2026.1760253 (PMC12960161; doi:10.3389/fvets.2026.1760253)
Supplement: Supplementary file 1 [file Table_1.docx]

1. Region

2. City

3. Sample Name / Farm ID

Herd Structure

4. Number of young stock (12 months)

5. Number of adult cows

Water Source

Codes:

- 1 = Municipal / public water supply
- 2 = Groundwater (well water)
- 3 = Spring water

6. What is the primary water source used on your farm?

Feeding System (Pasture Use)

Proportion of pasture in whole feed (Proportion_diet_concentrate)

- 1 = <25% pasture
- 2 = 25–50% pasture
- 3 = >50% pasture

7. What proportion of the diet comes from pasture?

Dog Ownership

8. Do you own one or more dogs on the farm?

- 0 = No
- 1 = Yes

Abortion History

9. Have you had abortions in your herd during the last 5 years?

- 0 = No
- 1 = Yes

Stray Dog Access to Pasture

10. Do stray dogs have access to your grazing areas?

- 0 = No
- 1 = Yes

Dog Feeding Practices

11. Dog feed (type / feeding practice)

- 1 = Canned/dry food
- 2 = Leftovers
- 3 = Raw meat
- 4 = Dog hunts rodents/birds

Farm Dog Access to Feed Storage

12. Do farm dogs have access to feed storage areas?

- 0 = No
- 1 = Yes
